# Liquid Biopsy as a Minimally Invasive Source of Thyroid Cancer Genetic and Epigenetic Alterations

**DOI:** 10.22088/IJMCM.BUMS.8.2.19

**Published:** 2019-05-29

**Authors:** Fatemeh Khatami, Bagher Larijani, Shirzad Nasiri, Seyed Mohammad Tavangar

**Affiliations:** 1 *Chronic Diseases Research Center, Endocrinology and Metabolism Population Sciences Institute, Tehran University of Medical Sciences, Tehran, Iran.*; 2 *Endocrinology and Metabolism Research Center, Endocrinology and Metabolism Clinical Sciences Institute, Tehran University of Medical Sciences, Tehran, Iran.*; 3 *Departments of Surgery, Tehran University of Medical Sciences, Shariati Hospital, Tehran, Iran.*; 4 *Departments of Pathology, Dr. Shariati Hospital, Tehran University of Medical Sciences, Tehran, Iran.*

**Keywords:** Liquid biopsy, ctDNA, circulating tumor cells, exosomes, mutation, methylation

## Abstract

In the blood of cancer patients, some nucleic acid fragments and tumor cells can be found that make it possible to trace tumor changes through a simple blood test called “liquid biopsy”. The main components of liquid biopsy are fragments of DNA and RNA shed by tumors into the bloodstream and circulate freely (ctDNAs and ctRNAs). Tumor cells which are shed into the blood (circulating tumor cells or CTCs), and exosomes that have been investigated for non-invasive detection and monitoring several tumors including thyroid cancer. Genetic and epigenetic alterations of a thyroid tumor can be a driver for tumor genesis or essential for tumor progression and invasion. Liquid biopsy can be real-time representative of such genetic and epigenetic alterations to trace tumors. In thyroid tumors, the circulating *BRAF* mutation is now taken into account for both thyroid cancer diagnosis and determination of the most effective treatment strategy. Several recent studies have indicated the ctDNA methylation pattern of some iodine transporters and DNA methyltransferase as a diagnostic and prognostic biomarker in thyroid cancer as well. There has been a big hope that the recent advances of genome sequencing together with liquid biopsy can be a game changer in oncology.

Liquid biopsy is the analysis of biomarkers through the patient’s body fluids (non-solid biological tissue) like cerebrospinal fluid, pericardial effusion, urine, and blood (1). It was about 150 years ago in 1869 that a pathologist, Thomas Ashworth, provided an evidence for the existence of circulating tumor cells (CTCs) in the blood of a metastatic prostate cancer patients (2). Those days his discovery was ignored but over the last decade, it has been discussed as the new and non-invasive source for solid tumor genes and cells. In fact, it has some remarkable advantages over the traditional tissue biopsy method because it has no risk, it is a minimally invasive method, it does not need surgery, and is a low-cost biopsy method (3). However, there is under the debate that it will take the place of tissue biopsy for cancer diagnosis or it will just support the result of tissue biopsy (4, 5). Similar to traditional tissue biopsy, it is not only a procedure for cancer diagnosis, but it also can be beneficially used for tracking tumor cells and mutations during treatment. It means that liquid biopsy can help discover the efficiency of a cancer treatment drug through multiple sampling during treatment, predicting the tumor relapse (6). While liquid biopsy-has been authenticated and approved by the Food and Drug Administration (FDA) as a beneficial prognostic method for various types of cancer, its clinical application is not yet widespread (7). In spite of the fact that liquid biopsy is mainly related to cancer diagnosis and management, there are some other occasions recruiting liquid biopsy like heart attack diagnosis (circulating endothelial cells (CECs)) and prenatal diagnosis, cell-free fetal DNA (cffDNA) extracted from maternal blood, isolation of protoporphyrin IX from blood samples of  atherosclerotic patients (8, 9).

The liquid biopsy approach in oncology has been mainly concentrated on the analysis of (CTCs, circulating tumor nucleic acids (ctNAs/ctDNA) and/or tumor-derived extracellular vesicles (exosomes), and tumor extra- chromosomal circular DNA (ecDNA) which have been shed from tumors, and their metastatic sites into the body fluids of cancer patients (10, 11). Liquid biopsies can bring new insights into the intra-tumor heterogeneity and genetic and epigenetic alterations responsible for metastases and treatment efficacy (11, 12). In thyroid malignancies, the liquid biopsy components can be important for its diagnosis and prognosis (figure 1). These genetic and epigenetic changes can be different in each individual and liquid biopsy concept can improve the personalized medicine approach (13, 14). 

**Fig. 1:**
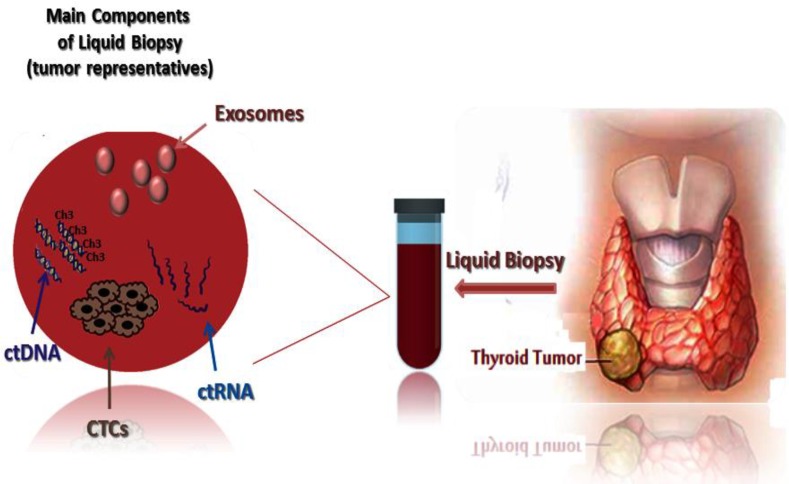
Liquid biopsy contains tumor representatives. The main components of liquid biopsy are: circulating tumor cells (CTCs) which should aggregate together to form CTC clustering in order to seed metastasis; circulating tumor DNA (ctDNA) in which both genetic (point mutations) and epigenetic (DNA methylation) can be detected; circulating RNA (ctRNA); and the last but not the least exosomes

## Thyroid cancer

Thyroid cancer is the most frequent endocrine neoplasia with increasing incidence in the past few decades (15-18). Thyroid cancers are epithelial driven tumors that typically arise from thyroid follicular cells including papillary thyroid carcinoma (PTC), follicular thyroid carcinoma (FTC), and anaplastic thyroid carcinoma (ATC). There is also another type of thyroid cancer called medullary thyroid carcinoma (MTC), originating from thyroid parafollicular (C) cells (19). PTC and FTC are considered as differentiated thyroid cancers (DTC) since they are well differentiated with indolent tumor growth. PTC represents 85-90% of entire thyroid cancer cases, FTC 5-10% thyroid cancers, and MTC up to5%. ATC as the rare one represents about 2% of thyroid cancers, and classically occurs in the aged patients and its incidence is associated directly with the age of patients (19). Thyroid cancer diagnosis is usually done through cytology and histopathology of thyroid tissue earning by fine needle aspiration (FNA) (20, 21). Histology and immunohistochemistry (IHC) are the usual diagnostic strategies with the highest positive predictive value of discrimination between neoplastic and non- neoplastic mass lesions (22-27). Classification and staging of thyroid cancers are based on the American Joint Cancer Committee TNM staging system. This system is according to the size and extent of the primary thyroid tumor (T), lymph node involvement (N), and distant metastases (M) (28). Management decisions for thyroid cancer consists of observation, surgery, radioiodine therapy, and pharmacotherapy. However, there are several controversies for optimal management approaches in patients with different types of thyroid cancer. The origin and molecular properties of thyroid cancers are the main factors that can determine disease progression and efficacy of response to the therapy. Consequently, new findings on the genetic and epigenetic of thyroid neoplasms can improve the identification of new disease biomarkers, and will result in improved thyroid cancer management. Research advances in the past decade have also uncovered additional genetic and epigenetic alterations as good candidates of personalized thyroid cancer management tool and therapeutic targets.

## Genetic of thyroid cancer

Thyroid cancer is the most usual neoplasm of endocrine system with an incidence of about 2-5 female and 1-2 male per 100,000 populations (17, 18, 29). The incidence of thyroid cancer has increased over the last recent years and in the United States, it has been reported that thyroid cancer incidence has changed to an annual rate of 5.4% in men and 6.5% in women from 2006 to 2010 (30).

Some genetic changes in thyroid cancer are suggested as the gate keeper or driver alteration that trigger thyroid tumorigenesis through activating metabolic pathways (31, 32). The mitogen-activated protein kinase (MAPK) pathway which is mediated by *ERK, JNK*, and *p38*, includes some components and phosphorylation events critical for cell transformation toward tumor formation. Modification of the *RAS-RAF-MEK-ERK-MAPK (RAS-MAPK)* pathway has often been described in several human cancers including thyroid cancers (33). Generally, extracellular growth factors stimulate the kinase cascade pathway by binding to receptor tyrosine kinases which finally leads to the transcription of genes that encode proteins for normal thyroid cell growth, proliferation, and differentiation. This pathway, especially *BRAF* mutations, is considered a potential therapeutic target for thyroid cancer treatment. Recently, some small-molecule inhibitors targeting the MAPK pathway through *BRAF *(vemurafenib, dabrafenib /tafinlar, and trametinib/ mekinist) have been developed and tested in some clinical trials (34-36). Most *RAS*-positive thyroid cancers have indeterminate cytology with low-grade follicular variant histology PTC and lymph node, and distant metastases are rare. It was suggested that total thyroidectomy would be done based on *RAS*-positive FNA results (37). RET (rearranged during transfection) is a receptor tyrosine kinase critical for the development of different human cancers, including PTC and MTC. The *RET* gene encodes for a tyrosine kinase transmembrane receptor expressing in a variety of neuronal cell lineages including thyroid C cells and adrenal medulla. *RET/PTC *rearrangement has been recognized for nearly two decades as one of the most common molecular alterations in PTC (38). Although still debated, recently it has been reported that *RET* gene expression may also occur in follicular thyroid cells. Therefore, RET protein has to turn out to be a gifted molecular target for thyroid cancer treatment (39).

The sodium-iodide symporter (NIS or SLC5A5) is a key plasma membrane protein that mediates active iodine uptake in thyroid, as the first step in the biosynthesis of iodine-containing thyroid hormones. Additionally, some other genetic alterations and rearrangements are candidate as the triggering thyroid cells to neoplasm formation including *CCDC6, NCOA4, PTEN, NRAS, CTNNB1, KRAS, NODAL, HRAS, PPARG, PAX8, APC, MEN1*(40, 41). Based on the targeted candidate genetic alterations several FDA approved components are suggested for thyroid cancer treatment (42-44). 

## Epigenetic of thyroid cancer

More than genetic changes there are some epigenetic alterations which can change the pattern of gene expression and trigger a normal thyroid cell to cancer cells. Epigenetics are heritable changes in gene expression with no direct changes of the DNA sequence, as a change in phenotype without a change in genotype (45). Epigenetic changes (DNA methylation, microRNAs (miRs) fluctuations, histone modifications, remodeling, post-translational modifications of chromatin, and nucleosome positioning) play important roles in thyroid tumorigenesis. Epigenetic silencing of many thyroid-specific genes has been detected in thyroid tumors (46, 47). Epigenetic alterations in oncogenes and tumor-suppressor genes are important for uncontrolled thyrocyte growth leading to the cell motility and invasiveness. Some recent studies indicated to the epigenetic mechanisms contributing to promoter hypermethylation and silencing of thyroid stimulating hormone receptor (*TSHR*) gene have been documented in thyroid carcinomas (48). There are some other candidate genes like  phosphatase and tensin homolog (*PTEN*), *Ras association domain-containing protein 1 *(*RASSF1A*)*, *tissue inhibitor of metalloproteinase-3 (*TIMP3*), sodium-coupled solute transporter (*SLC5A8*), death-associated protein kinase (*DAPK*), and retinoic acid receptor β2 (*RARβ2*) (49, 50). Methylation of Na+/I− symporter (*NIS*), the promoter of the TSH receptor is associated with the suppression of iodide- metabolizing molecules and results in the loss of impact of radioiodide therapy. Circulating O6- methylguanine- DNA methyl transferase (*MGMT*) promoter methylation can be suitable candidates as PTC biomarkers (51). *PTEN* gene encodes a phosphatase that dephosphorylates phosphatidylinositol -3,4,5-trisphosphate which is involved in the arrest of the signaling of the phosphatidylinositol 3-kinase (PI3K)/Akt pathway. The *RASSF1A* gene encodes a protein similar to the RAS effector proteins, and interacts with connector enhancer of kinase suppressor of ras 1 (*CNKSR1*), Death-associated protein 6 (*DAXX*), GTPase HRas* (HRAS)*. The protein inhibits the accumulation of cyclin D1 resulting in cell cycle arrest. Among the different thyroid tumors, methylation of *RASSF1A *and *PTEN* more often happened in FTC. There is also a link between *BRAF* mutation and aberrant methylation of some genes involved in thyroid malignancies. By way of illustration, tissue inhibitor of matrix metalloproteinase-3 (*TIMP3*), death-associated protein kinase* (DAPK*), and retinoic acid receptor β2 (*RARβ2*) aberrant methylation and *BRAF* mutation has been reported in PTC. Promoter hypermethylation of these genes in PTC was completely correlated with extra-thyroid extension (ETE), lymph node metastasis, and advanced disease stages (III and IV).In addition, silencing of *TIMP3 *gene through its promoter methylation may play a distinctive role in *BRAF *mutation-promoted invasiveness and progression of PTC.

The other epigenetic alterations of eukaryotic cells are histone modifications which happen as the covalent post-translational modifications (PTM) of histone proteins (methylation, phosphorylation, acetylation, ubiquitynation, and sumoylation) (52). It is shown that some of these histone methylation and phosphorylation are important in the regulation of transcription by thyroid hormone receptor. Generally, histone modifications disturb normal chromatin conformation and nucleosome positioning, and change the gene transcription profile of a normal cell (53, 54). Histone acetylation and deacetylation are controlled by histone acetyltransferases (HATs) and histone deacetylases (HDACs) which are targeted by several FDA approved epidrugs in thyroid cancers (55). 

MiRs and long non-coding RNAs (lncRNAs) are non-coding RNA molecules in charge of the mammalian gene expression controlling as epigenetic regulators (56). It is suggested that miRs expression in normal thyroid tissue versus neoplastic tissue has 32% up-regulated miRs and 38% and down-regulated miRs (57). Two histone deacetylase inhibitors, trichostatin A and vorinostat, made miR-129-5p overexpressed, and caused cell death in PTC and ATC (58). Overexpressed miR-497 blocked thyroid cancer cell proliferation, migration, and invasion in vitro and introverted tumorigenesis in vivo. In fact, in thyroid cells, miR-497 plays a role through interaction with oncogene brain-derived neurotrophic factor (*BDNF*) (59). MiR-146b stimulates PI3K/AKT pathway which results in thyroid cancer progression targeting PTEN (60). Overexpression of many suggesting miRs were established in major types of thyroid tumors (61). A meta-analyses indicated the deregulation of miRs like miR-146b, miR-221, miR-222, and miR-181b in PTCs (62). Three miRs, -146b, -221, and -222, are in the same way overexpressed in FTC, Hürthle cell thyroid carcinomas, and ATC (63, 64), whereas miR-197 and miR-346 are increased especially in FTC. A group of miR-17-92 are particularly up-regulated in anaplastic thyroid cancer, so this cluster can trigger the oncogenic process (65). 

## Liquid biopsy in thyroid cancer

A liquid biopsy as a simple and non-invasive tool enables scientists to trace all solid tumor genetic and epigenetic alterations through a simple blood sample. So, all thyroid tumors molecular characteristics can be taken into account for liquid biopsy. Over the last few years, some studies have reported the importance of liquid biopsy in diagnosis, prognosis and personalized medicine of thyroid cancer (66). *BRAF* mutation in blood, circulating *BRAF* mutation, the most common genetic alterations of differentiated PTC and ATC has been suggested several times as a beneficial tool for early detection (67). A clinical trial phase II in Philadelphia indicated the antitumor efficacy of vemurafenib in PTC patients who were *BRAF*^V600E^*-*positive and had never been treated with multi-kinase inhibitors (68, 69). In fact,  circulating *BRAF*^V600E^  is detectable in the blood of PTC patients, and can therefore be a biomarker for prognosis, surveillance, and clinical decision making (70). Blood *BRAF*^V600E^ levels in both an established animal model and patients with all stage of thyroid cancer harboring *BRAF*^V600E^-positive tumors can be assessed to predict the response to treatment (71). In spite of the fact that most studies indicated several advantages of liquid biopsy over fine-needle aspiration biopsy for thyroid cancer diagnosis, there are still some controversies (72). ctDNA*RET M918T* in patients with advanced MTC has prognostic significance for overall survival and monitoring response to treatment (73). More than ctDNA mutation there are some pieces of evidence of ctDNA methylation detection as remarkable diagnostic and prognostic thyroid cancer biomarkers. A panel of circulating *SLC5A8* and *SLC26A4* hypermethylation and *BRAF*^V600E^ analysis was shown as an easy, reproducible, and non-invasive tool for thyroid cancer diagnosis (74). The recent comprehensive human cell-type methylation atlas has revealed the origins of circulating cell-free DNA in health and disease and proposes a procedure which can be easily adapted to ctDNA in many settings (75). In addition, plasma cell-free DNA methylome analysis was suggested as the sensitive tool for several solid tumor detection and classification including thyroid malignancies (76). The methylation landscape is referred to methylscape, and can be seen in several cancer types, so it might work as a general cancer biomarker (77). The consequence of the presence of methylcytosines on the physicochemical properties of DNA was inspected by Sina et al. who tried to detect the methylscape biomarker (77). They established that DNA polymeric trait is strongly linked to the differential patterning of methylcytosine and can change DNA solvation and DNA-gold affinity between cancerous and normal genomes. It is suggested that methylscape changes can be settled as a very sensitive and selective electrochemical or colorimetric one-step assay for cancer detection. 

Unfortunately, limited reports are presented on the expression and clinical significance of circulating miRs in thyroid cancer. Circulating miR-146b-5p, miR-221-3p, and miR-222-3p in PTC were shown to increase in thyroid cancer patients in comparison with healthy individuals (78). In addition, miR-222 and miR-146b may discriminate PTCs from benign nodules (79). The amount of miR-21 in FTC patients is increased significantly in patients with benign nodules or PTC, contrary to the miR-181a that is up-regulated in PTC patients in comparison with those with FTC (80). The follow-up of thyroid cancer patients showed that circulating levels of miR-146a-5p, miR-146b-5p, miR-221-3p, and miR-222-3p decreased significantly in PTC patients who underwent thyroidectomy surgery (78). So they can be important as the liquid biopsy elements which indicate the efficacy of the treatment. Two circulating miR-940 or miR-486-5p can be good predictive biomarkers for the early diagnosis of spontaneous abortion (SA) in patients with subclinical hypothyroidism (81).

In the field of histone modification and nucleosome positioning, it seems that since ctDNAs are small fragmented DNA molecules, so they can not represent information about histones and nucleosomes. However, there are some shreds of evidence that circulating nucleosomes can be biomarkers in an exciting novel extent of research to allow both the early detection of cancer and monitoring of treatment response (82).

According to the fact that the ctDNAs are released from tumor cells after cell apoptosis and necrosis, the length of ctDNA is corresponding to nucleosomes (~147 base pairs) and chromatosomes, nucleosome plus linker histone with about 167 base pairs. In eukaryotic cells, there are proteins called histones as a family of small, positively charged proteins termed H1, H2A, H2B, H3, and H4. DNA molecule is negatively charged, due to the phosphate groups in its phosphate-sugar backbone, so histones bind with DNA very compactly and make nucleosome. A nucleosome is a basic unit of DNA packaging in eukaryotes, made up of a core particle consisting of approximately 146 base pairs of DNA wrapped in 1.67 left-handed superhelical turns around a histone octamer, which is about equal length of ctDNA. Core particles are connected by stretches of "linker DNA", which can be up to about 80 base pair long. Linker histones such as H1 and its isoforms are involved in chromatin compaction and sit at the base of the nucleosome near the DNA entry and exit binding to the linker region of the DNA. Nucleosome positioning varies between cells based on gene expression profile and epigenetic changes of histones and DNA, and is called “nucleosome footprints”. Deep sequencing of ctDNA isolated from circulating blood plasma and generating maps of genome-wide *in vivo* nucleosome occupancy, showed that ctDNA fragments harbor footprints of transcription factors (83). The ctDNA nucleosome occupancies associate with the nuclear architecture, gene structure, and expression observed in normal cells versus tumor cells, so they could specify the tumor cell-type of origin (83). In fact, nucleosome footprints can be used to find the origin of tumor and cell types funding to ctDNA in pathological states such as thyroid cancer.

## Future perspective of liquid biopsy in thyroid cancer management

Several studies evaluated new approaches for thyroid cancer detection based on the genetic and epigenetic alterations of tumor cells. New insight of liquid biopsy together with recent advances of molecular biology techniques like next-generation sequencing (NGS), genome-wide association studies (GWAS), epigenome-wide association studies (EWAS), single-cell DNA methylome sequencing, can be recruited by the oncologist to early diagnosis and tracing of the treatment efficacy in the minimally invasive way. Linking ctDNA information with protein markers of thyroid tumor can offer some information about where cancer may be found and where the origin of the tumor was (84). Very recently, some interesting researches have indicated that blood-based liquid biopsies can offer a minimally invasive alternative to identify cellular and molecular signatures that can be used as biomarkers to detect early-stage cancer, predict disease progression, longitudinally monitor response to chemotherapeutic drugs, and provide personalized treatment options (85). Preliminary research of fluid-harvested tumor materials have been reported, but today propagation of tumor cells in non-blood body fluids with the term of mobile tumor cells  is gaining importance (86).
